# Transformer architecture for diagnosing schizophrenia disabilities through EEG analysis

**DOI:** 10.3389/fphys.2026.1663906

**Published:** 2026-07-01

**Authors:** Nizar Alsharif, Nadhem Ebrahim, Abdullah H. Al-Nefaie, Zeyad A. T. Ahmed, Theyazn H. H. Aldhyani

**Affiliations:** 1 Department of Computer Science, Al-baha University, Albaha, Saudi Arabia; 2 Department of Computer Science College of Engineering, University of Akron, Akron, OH, United States; 3 Department of Quantitative Methods, School of Business, King Faisal University, Al-Ahsa, Saudi Arabia; 4 Faculty of Data Science and Information Technology, INTI International University, Nilai, Negeri Sembilan, Malaysia; 5 Applied College, King Faisal University, Al-Ahsa, Saudi Arabia

**Keywords:** deep learning, disabilities, mental health, schizophrenia, transformers

## Abstract

**Introduction:**

Schizophrenia (SZ) presents significant diagnostic challenges in clinical practice.

**Methods:**

In this study, we explore novel deep learning approaches for the automated detection of this severe psychiatric disorder through electroencephalography (EEG) analysis. Using a publicly available dataset of EEG recordings from 14 SZ patients and 14 healthy controls, we developed a robust processing pipeline that includes bandpass filtering (0.5–45 Hz), artifact removal through Independent Component Analysis, and signal enhancement via wavelet transformation. Our feature extraction approach captured both temporal characteristics, including statistical measures such as mean, standard deviation, skewness, and kurtosis, as well as spectral properties, including power distributions across the delta, theta, alpha, beta, and gamma bands. By combining the F-score method, based on Analysis of Variance (ANOVA), Mutual Information assessment, and random forest techniques, we identified 27 highly discriminative features from the original set of 171 extracted features. We developed and evaluated two novel architectures: an LSTM-based model and a Transformer-based model. Both incorporated attention mechanisms and multi-scale temporal convolutional networks to effectively capture the complex patterns in EEG signals.

**Results:**

While the LSTM model performed strongly with 95.65% accuracy (± 0.16%), 96.28% sensitivity (± 0.59%), and 95.26% specificity (3.84%), our Transformer-based model achieved even more impressive results: 98.20% accuracy (± 0.20%), 98.11% sensitivity (± 0.28%), and 98.12% specificity (± 0.40%) by using k-fold cross-validation. The Transformer-based was achieved 76.95% in Leave-One-Subject-Out (LOSO) cross-validation. These findings highlight the potential of transformer architectures to detect the subtle neurophysiological markers of schizophrenia in EEG recordings.

**Discussion:**

Our approach could eventually provide clinicians with an objective tool to support earlier and more accurate diagnosis of schizophrenia, potentially improving treatment outcomes through timely intervention.

## 1 Introduction

Schizophrenia (SZ) is a life-altering, chronic brain disorder. Symptoms of active schizophrenia include disorganized speech, hallucinations, cognitive impairment, and a lack of motivation. The good news is that treatment may alleviate most symptoms of schizophrenia and reduce the likelihood of a recurrence (Aslan and Akin, 2022; Dabiri et al., 2022; Luvsannyam et al., 2022; Teixeira et al., 2023). It is a psychiatric disorder that affects roughly 20 million people worldwide. It entails atypical brain development, leading to symptoms like paranoia and auditory hallucinations (Thompson et al., 2001; Van Essen et al., 2012). People with schizophrenia are at a greater risk of early mortality than healthy persons due to the elevated incidence of medical disorders. SZ affects both sexes. However, data indicates that men may manifest the illness earlier. Researchers and scientists are examining genetics and contemporary methodologies to comprehend the brain’s architecture (Thompson et al., 2001; Van Essen et al., 2012).

Schizophrenia affects roughly 1% of the world’s population at some point in their life. This amounts to an estimated 20 million or more people worldwide living with schizophrenia. Considered one of the leading causes of impairment worldwide, the average worldwide incidence rate of schizophrenia is also estimated to be around 15 cases per 100,000 people annually. About 1% of the world’s illness load is related to the impairment linked to schizophrenia (Angst, 1998; Velligan and Rao, 2023).

SZ stands as a profoundly severe psychological disorder, exerting devastating effects on both the brain and the daily activities of affected individuals (Sharma et al., 2023). The disorder is associated with disruptions in initial brain growth, leading to diverse symptoms encompassing hallucinations, cognitive issues, motivational problems, and disordered thought patterns (Sahu et al., 2023). While the exact cause of this neural disorder remains unknown, neuroscientists propose a multifaceted origin involving the interplay of genetic factors and various environmental influences (Sahu et al., 2023; Sharma et al., 2023). Approximately 21 million people globally contend with SZ, with onset typically occurring at ages 18 and 25 for women and men, respectively (Iyortsuun et al., 2023; Shoeibi et al., 2023).

Diagnosing SZ poses a formidable challenge due to the disorder’s heterogeneity and the absence of specific biomarkers. Clinical examination involves evaluating physical, psychiatric, and psychological indicators through various tests, including blood tests and medical imaging [WHO]. The DSM-5 has introduced specific criteria to aid specialists in SZ diagnosis, departing from earlier practices that relied on single symptoms for diagnosis [WHO, NIH].

Efforts to detect SZ have extended into virtual reality tasks, revealing potential in measuring cognitive flexibility. Additionally, comorbid disorders like schizoaffective disorder further complicate the diagnostic landscape (Aslan and Akin, 2022). Neuroimaging modalities, including structural MRI, diffusion tensor imaging (DTI), electroencephalography (EEG) (Zheng et al., 2019; Pei et al., 2022; Pei et al., 2025), magnetoencephalography (MEG), and functional MRI (fMRI), have been employed to gain insights into brain structure and function (Han et al., 2016; Pei et al., 2021; Sadeghi et al., 2022; Wang et al., 2024). Despite the benefits of these modalities, challenges such as noise, artifacts, and limited real-time monitoring persist. As a result, computer-aided diagnostic systems (CADS) that use advanced image processing, machine learning (ML), and deep learning (DL) techniques have been developed to improve the accuracy and efficiency of SZ diagnosis. These technological advancements signify a promising direction in overcoming the complexities associated with SZ diagnosis (Kafai and Eshghi, 2017; Azevedo, 2019).

EEG has proven a remarkable impact in treating many mental health disorders in recent years. Several algorithms were employed for automated EEG analysis, considering technical developments, data availability, the robustness of ML algorithms, and the affordability of high-performance computers (Kafai and Eshghi, 2017). The literature widely employs various classification techniques, including Support Vector Machines (SVM) (Kumar and Kumutha, 2020; De las Casas et al., 2021) and neural networks (Murugan et al., 2019; de Mendonça Mesquita et al., 2021; Lai et al., 2021). K-Nearest Neighbor (KNN) (Cortes-Briones et al., 2022; Sadeghi et al., 2022) and Adaptive Boosting (Adaboost) (Barros et al., 2021; Sadeghi et al., 2022) were other techniques proven viable for data categorization.

SZ is marked by hallucinations, delusions, and disorganized speech, leading to social or occupational dysfunction. Diagnosis requires excluding organic causes and conditions like dementia or delirium. Treatment involves non-pharmacological interventions, such as cognitive-behavioral therapy, to help patients manage symptoms and achieve optimal psychosocial functioning. Pharmacological treatment, focusing on neurotransmitter modulation, remains the primary approach to managing symptoms. MRI and fMRI are the most effective techniques for detecting SZ; however, they are costly and not scalable.

The use of artificial intelligence (AI) algorithms in the medical field has been notable since the emergence of modern computers. As computational power has advanced and medicine has become more complex, the intersection of AI and medicine has witnessed increased collaboration, presenting unexplored potential. Advancements in AI models are revolutionizing the analysis and processing of extensive biomedical data, enhancing predictive capabilities in research and healthcare delivery. These technologies have been extensively employed in creating predictive models for various medical purposes, transforming clinical decision-making and diagnoses (Zhang, 2020; Lai et al., 2021; Luján et al., 2021; Aslam et al., 2026; Rasool et al., 2026).

This research primarily focuses on a distinctive brain connection matrix that combines data from multiple sources to improve schizophrenia detection through EEG analysis. Redundancy occurs in symmetric connection matrices because traditional methods of using EEG data to identify schizophrenia often rely on just one measure of connectivity. To solve this problem, the present study utilized attention mechanisms and multi-scale temporal convolutional networks to adeptly capture the intricate patterns in EEG signals, thereby improving classification via DL models. Detecting schizophrenia early by analyzing EEG data with classification algorithms is essential, as it helps doctors precisely identify the issue.

The main contribution of the proposed system is to develop a comprehensive feature selection methodology combining statistical testing, information theory, and ensemble learning to identify 27 highly discriminative EEG features from 171 candidates. We designed novel dual-branch neural architectures that simultaneously process raw temporal data and statistical features, enabling more comprehensive signal analysis than traditional approaches. Our system employs advanced attention methods that focus on key areas and frequency ranges in the brain, illustrating how schizophrenia impacts the brain. Incorporating multi-scale temporal convolutional networks effectively captures EEG patterns across different time resolutions, addressing the complex nature of schizophrenia-related neurophysiological abnormalities. The experimental results demonstrate that our transformer-based model excels at identifying brain activity changes associated with schizophrenia, achieving an accuracy of 98.30%. This performance surpasses that of the LSTM-based model, setting new standards for the automatic detection of schizophrenia using EEG signals.

## 2 Related works

Many researchers and scientists have lately tried to diagnose schizophrenia disabilities using EEG data. Several hand-engineered ML and DL methods have emerged to address this challenge. This section systematically reviews the present techniques and their advantages and drawbacks.

Lei Zhang (2020) proposed ANN architecture wherein EEG data were captured. ANN achieved a maximum categorization accuracy of more than 98%. These results indicate a promising possibility for developing a comprehensive diagnostic tool for SZ, incorporating both subjective and objective assessments based on EEG data. Siuly et al. (Aslam et al., 2026) explored the automatic use of deep residual networks (ResNet) for SZ discrimination from EEG data. Used average filtering to minimize noise in the EEG signals. Then, a ResNet was used to automatically extract relevant features from the recordings. At the end, the retrieved features were classified using a softmax layer. Automatic SZ identification using EEG data using DL models was shown by C. Barros (Rasool et al., 2026) with possible diagnostic ramifications; this work demonstrates that DL methods help detect SZ with impaired auditory processing. The RF model used by Buettner et al. (2019) used the RepOD dataset (EEG in schizophrenia). Using a differential diagnostic system with this technology will improve the efficiency, precision, and affordability of intensive care unit therapies.

In S. Krishnan et al. (2020) integrated individual EEG data into intrinsic mode functions (IMFs) signals. An approximation of the IMF signal’s unpredictability was obtained by computing its entropy. Numerous ML classifiers were trained using a feature matrix constructed from the IMF signal’s entropy values. The radial basis function support vector machine (SVM-RBF) outperformed the other methods, achieving an F1-score of 93% and the highest accuracy across all 95 attributes. Using EEG data segmentation, normalization, filtering, and early SZ detection, Shoeibi et al. (2024) investigated these topics. A vector-based transformer design with various activation functions was used to extract features from preprocessed electroencephalogram data. In their innovative method, Bagherzadeh et al. (2022) analyzed the EEG data using the transfer entropy (TE) methodology to find the effective connection matrix. Compared to their pre-trained CNN counterparts, the hybrid CNN-LSTM models performed better.

Chang et al. (2021) used Gaussian process regression analysis to study how the brains of HC and SZ patients connect while they performed the mismatch negativity task. Brain connectivity was observed in both HC and SZ individuals during the mismatch negativity procedure. The relationship between mismatch negativity and Global Assessment of Functioning (GAF) scores showed correlation coefficients of 0.73, an R-squared value of 0.53, and a p-value of 0.0006. observed for the relationship between mismatch negativity and Global Assessment of Functioning (GAF) scores. A case of SZ diagnosed via ML was detailed by Febles (Santos Febles et al., 2022). Across the board, the multiple kernel learning (MKL) classifier achieved 83% accuracy on the task. Classification accuracy increased to 86% after the Boruta feature selection approach was implemented. The delay and loudness of the auditory P300 paradigm were the most important parameters for classification.

Oh et al. (2019) constructed two eleven-layer CNN models to classify the original EEG data. Sridhar et al. (2021) introduced two long short-term memory (LSTM) designs and two channels, FP1 and FP2, to diagnose schizophrenia. For schizophrenia classification, Chandran et al. (2021) also used LSTM. The LSTM model is fed 6790 features retrieved using three non-linear methods: variance, approximation entropy, and Katz fractal dimension. Another approach to identifying schizophrenia is to combine CNN and LSTM models. Shoeibi et al. (2021) assessed various traditional and DL methods. Their method involves applying L2 and z-score adjustments to separate the EEG data into 25-second trials. The most precise results were obtained by training 1D-CNN-LSTM with ReLU activation. Sharma and Joshi (2021) proposed a CNN and LSTM model-based approach. Supakar et al. (2022) and Sairamya et al. (2022) used the RLNDiP method to extract differentiating features from the time-domain (TD) and time-frequency-domain (TFD) of EEG data to build an LSTM model that could distinguish between EEG signals from healthy individuals and those from individuals with schizophrenia. What does the ANN model consider as its dominant features?

**Table 1** shows a few of these system.

**Table 1:** Existing systems.

Authors	Models	Accuracy
Shoeibi et al. (2023)	CNN-LSTM	81.22%
Devia et al. (2019)	LDA	71
Buettner et al. (2019)	RF	80
WeiKoh et al. (2022)	KNN	97.20
Khare et al. (2023)	CNN	97.4
Yang et al. (Ko and Yang, 2022)	VGGNet	93.20
Kim et al. (2021)	SVM	74.64
Park et al. (2020)	LR,SVM	80.4
Santos Febles et al. (2022)	SVM	83
De Rosa et al. (2022)	RF	95
Chin et al. (2018)	ANN	81.25
Siuly et al. (Aslam et al., 2026)	EBT	89.59
Oh et al. (2019)	CNN	89
Sharma and Joshi (2021)	CNN-LSTM	99
Singh et al. (Agarwal and Singhal, 2023)	CNN-LSTM	98.56
Phang et al. (2019a)	DBN	95

## 3 Methodology

**Figure 1:**
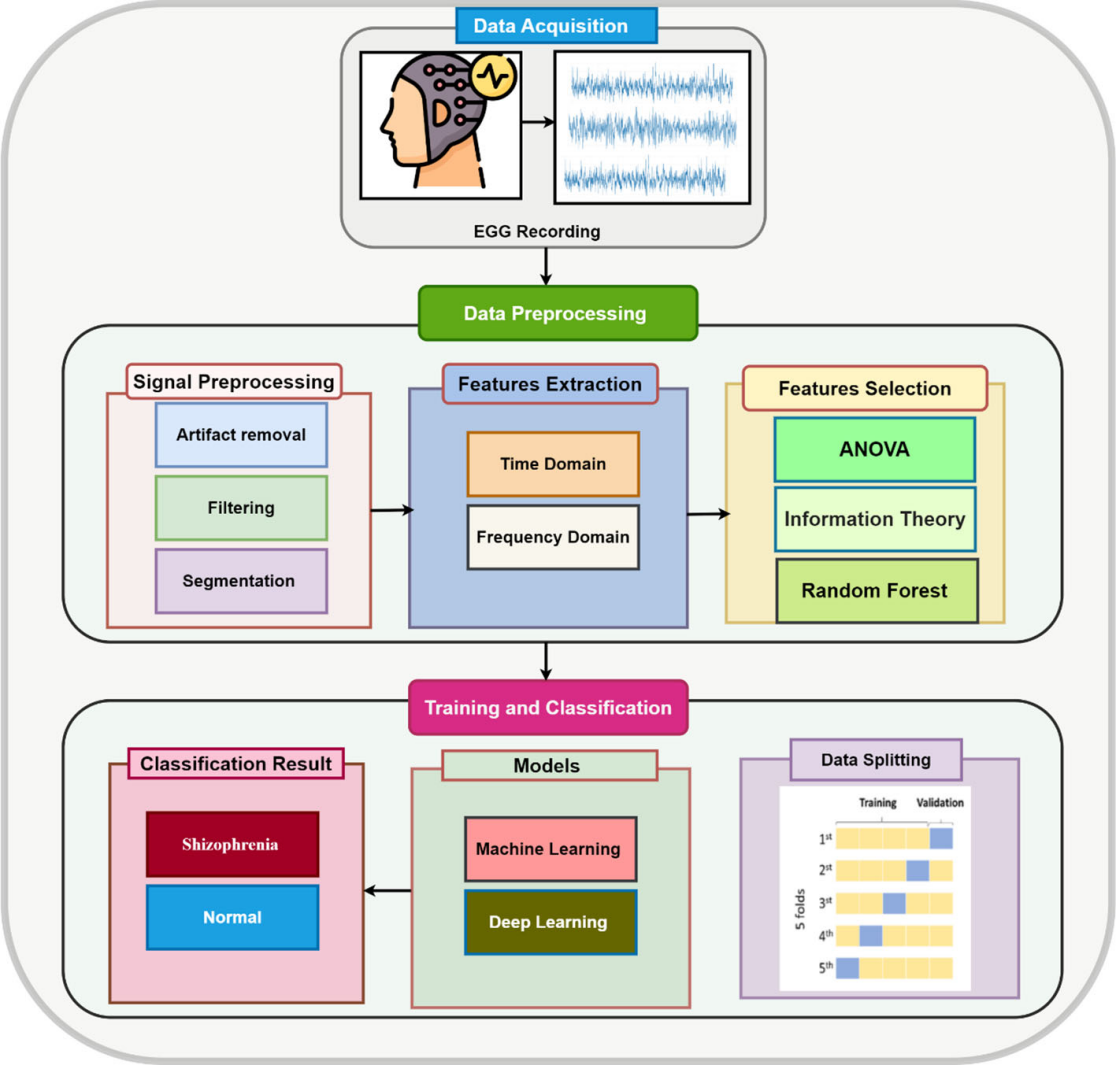
Framework of the proposed system.

**Figure 1** shows the proposed method for EEG-based schizophrenia detection, which follows a clear step-by-step process with five main stages. First, we use publicly available EEG datasets that include recordings from people with schizophrenia and healthy controls, ensuring good data quality and consistency. Second, we apply thorough signal preprocessing techniques, such as filtering and artifact removal, to improve the signal quality. Third, we extract important features from the cleaned EEG signals, capturing both time-based and frequency-based information. Fourth, we use advanced feature selection methods to pick the most useful features for classification. Finally, we create, and train innovative deep learning models specifically designed for EEG signal analysis and classification.

### 3.1 Dataset discretion

The foundation of our study rests on the publicly available EEG dataset published by Olejarczyk & Wojciech (Phang et al., 2019a). This carefully curated dataset comprises EEG recordings from 14 patients diagnosed with paranoid schizophrenia and 14 healthy control subjects. The EEG data were recorded using the standard 10–20 montage system, incorporating 19 EEG channels: Fp1, Fp2, F7, F3, Fz, F4, F8, T3, C3, Cz, C4, T4, T5, P3, Pz, P4, T6, O1, and O2. All recordings were conducted with a sampling frequency of 250 Hz, with the reference electrode strategically positioned between Fz and Cz electrodes to ensure optimal signal quality and consistency across all recordings. **Table 2** presents the details of the dataset.

**Table 2:** describes the dataset participants.

Category	Total subjects	Gender	Age (Mean ± SD)
HC	14	Males: 7, Females: 7	27.75 ± 3.15 years
SZ	14	Males: 7, Females: 7	28.1 ± 3.7 years
Total	28		

**Figure 2:**
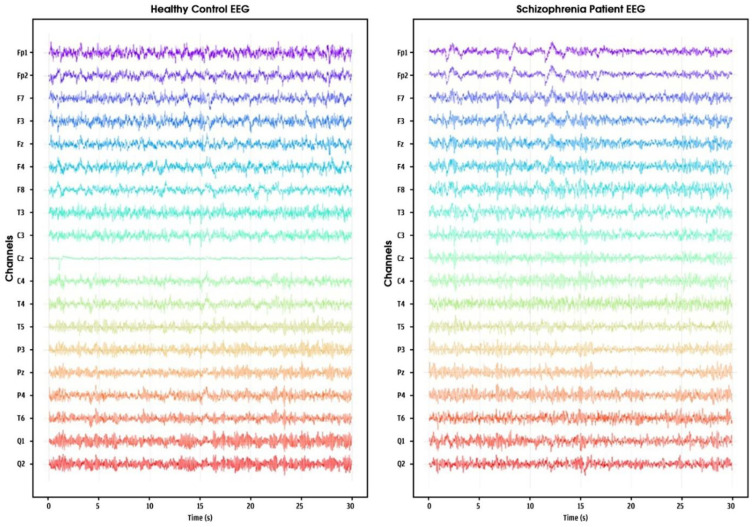
Comparative EEG waveform analysis of healthy controls vs. schizophrenia patients.

**Figure 2** shows sample EEG recordings from the original dataset, comparing brain activity patterns of healthy controls and schizophrenia patients. The samples include recording h01.edf from a healthy control and s01.edf from a schizophrenia patient, using the standard 10–20 montage system with 19 channels. Each recording represents a 30-second segment sampled at 250 Hz. The comparison highlights characteristic differences in spectral power distributions across frequency bands between the two groups. The healthy control displays consistent rhythmic patterns with stable amplitudes across all electrode sites. Alpha rhythms are well-defined, especially in occipital regions (O1, O2), and there is excellent symmetry between corresponding hemispheres. **Figure 2A** shows the healthy recording, which demonstrates the expected regular and organized electrical activity typical of normal brain function. In contrast, the schizophrenia patient’s recording shows increased variability in signal amplitude, particularly in frontal electrodes (Fp1, Fp2). The patterns are more irregular, with intermittent disruptions in normal rhythmic activity, as seen in **Figure 2B**. There is reduced alpha power in the occipital regions, increased activity in midline channels (especially at Cz), and more noticeable asymmetry between hemispheres. These features align with common neurophysiological abnormalities observed in schizophrenia.

These EEG differences reflect the underlying neural dysregulation and cortical connectivity disturbances in schizophrenia. The abnormalities in rhythmicity, amplitude, and symmetry provide objective neurophysiological markers that complement clinical assessment and may contribute to our understanding of the disorder’s pathophysiology.

### 3.2 Signal preprocessing

Preprocessing EEG signals is an essential first step in our method for detecting schizophrenia. Raw EEG recordings often include various kinds of noise and artifacts that can hide the true brain activity patterns. Our preprocessing process involves three main stages: bandpass filtering to focus on relevant frequency ranges, Independent Component Analysis (ICA)-based artifact removal to clean the signals, and wavelet-based enhancement to boost signal quality. These carefully chosen techniques work together to ensure that the following analysis is based on clean, high-quality EEG data that accurately reflects the brain’s electrical activity (Bagherzadeh et al., 2022; Abdur Rasool et al., 2025; Aslam et al., 2025).

#### 3.2.1 Bandpass filtering

EEG signals from 19 electrodes (10–20 system), sampled at f_s_ = 250 Hz, were bandpass filtered between f_L_ = 0.5 Hz and f_H_ = 45 Hz using a Butterworth filter. A 3rd-order lowpass prototype was specified, which yields an effective 6th-order bandpass filter after the standard lowpass-to-bandpass frequency transformation. The filter was applied bidirectionally (forward and reverse) to achieve a zero-phase response, which preserves the temporal structure of EEG components and avoids phase-induced distortion of event-related activity.

The lower cutoff at 0.5 Hz attenuates slow drifts and DC offsets, while the upper cutoff at 45 Hz suppresses 50 Hz power-line interference and high-frequency muscle artefacts while preserving the delta (0.5–4 Hz), theta (4–8 Hz), alpha (8–12 Hz), beta (12–30 Hz), and low-gamma (30–45 Hz) bands previously associated with schizophrenia-related neural activity (Dabiri et al., 2022; Luvsannyam et al., 2022). The Butterworth design was selected for its maximally flat passband response, which minimizes in-band amplitude distortion at the cost of a more gradual transition-band roll-off compared with Chebyshev or elliptic alternatives.

The squared magnitude response of an *N*-th order Butterworth bandpass filter is given by:

(1) $$|H(\omega ){|}^{2}=\frac{1}{1+{\left(\frac{\omega^{2}-\omega_{L}\omega_{H}}{\omega (\omega_{H}-\omega_{L})}\right)}^{2N}}$$

where ω_L_ = 2πf_L_ and ω_H_ = 2πf_H_ are the angular cutoff frequencies. With N = 3, f_L_ = 0.5 Hz, and f_H_ = 45 Hz, the filter passes the EEG frequency range of interest while attenuating out-of-band.

#### 3.2.2 Artifact removal

After initial filtering, we use Fast Independent Component Analysis (FastICA) to remove artifacts. This crucial step helps identify and eliminate common EEG artifacts, particularly those arising from eye movements, muscle activity, and electrical interference. The ICA process decomposes the EEG signals into independent components, allowing us to identify and remove artifact-related components before reconstructing the clean signal. The mathematical model expresses the observed data as a linear mixture of independent sources:

(2) X=AS

Where *X* is the observed data matrix, *S* contains the independent sources, and *A* is the mixing matrix. Fast ICA iteratively finds the unmixing matrix *W* such that *S = WX* by maximizing non-Gaussianity. This is achieved by approximating negentropy:

(3) $$J(y)\approx {\left[E\{G(y)\}-E\{G(v)\}\right]}^{2}$$

Where *G* is a non-quadratic function and *v* is a Gaussian variable. The algorithm uses fixed-point iteration:

(4) $$w^{+}=E\{xg(w^{T}x)\}-E\{g^{\prime }(w^{T}x)\}w$$

(5) $$w=w^{+}/|w^{+}|$$

This allows for identifying and potentially removing artifact components without significantly affecting the underlying neural signals.

#### 3.2.3 Wavelet-based signal enhancement

The final preprocessing stage utilizes the Adaptive Wavelet-Based EEG Enhancement with Residuals (ALEWER) technique. This advanced method employs a discrete wavelet transform with db4 wavelets across five decomposition levels. The ALEWER process adaptively enhances the EEG signals by performing wavelet decomposition, followed by intelligent coefficient thresholding for noise removal, and finally, reconstructing the improved signal. This process of ALEWER first transforms the signal into the wavelet domain using Daubechies-4 wavelets, creating a multi-resolution representation:

(6) W=ΨX

Where *Ψ* is the wavelet transformation matrix, for each set of wavelet coefficients, the algorithm calculates log-energy values:

(7) $$E(c)=\log_{10}(\sum c^{2})$$

Next, we normalize these energy values.

(8) $$E_{\text{norm }}=\frac{E-E_{min}}{E_{max}-E_{min}}$$

Coefficients with normalized energy below the threshold are zeroed out through adaptive thresholding:

(9) $$c_{\text{selected }}=\{\begin{array}{cc} c & \text{ if }E_{\text{norm }}\geq \text{ threshold }\\ 0 & \text{ otherwise } \end{array}$$

The filtered signal is then reconstructed through an inverse wavelet transform:

(10) $$\hat{X}={\text{Ψ}}^{-1}W_{\text{selected }}$$

This process effectively removes low-energy components typically associated with noise while preserving meaningful neural activity.

### 3.3 Feature extraction

Feature extraction transforms preprocessed EEG signals into quantifiable characteristics that can distinguish between healthy and schizophrenic patients. We extract both temporal features (statistical measures of the signal) and spectral features (power in different frequency bands) to capture the distinct patterns of brain activity. This dual approach ensures that we identify both time-based variations and frequency-specific changes that may indicate the presence of schizophrenia.

#### 3.3.1 Temporal domain features

Our temporal analysis computes statistical measures that characterize the signal’s amplitude distribution and variability. These include the mean, which represents the central tendency of the signal, and the standard deviation, which quantifies the variation in the signal’s amplitude. We also calculate higher-order statistics, specifically skewness and kurtosis, which provide insights into the signal’s asymmetry and peakiness, respectively. The following mathematical equation represents the Temporal Domain Features.

Mean

(11) $$\mu =\frac{1}{N}\sum_{i=1}^{N}x_{i}$$

Standard Deviation

(12) $$\sigma =\sqrt{\frac{1}{N}\sum_{i=1}^{N}{\left(x_{i}-\mu \right)}^{2}}$$

Skewness

(13) $$\text{γ}=\frac{1}{N}\sum_{i=1}^{N}{\left(\frac{x_{i}-\text{μ}}{\text{σ}}\right)}^{3}$$

Kurtosis

(14) $$\kappa =\frac{1}{N}\sum_{i=1}^{N}{\left(\frac{x_{i}-\mu }{\sigma }\right)}^{4}-3$$

The implementation extracts these features for each channel, creating a comprehensive representation of signal characteristics across the entire electrode montage. This multi-channel approach enables the capture of both global and localized abnormalities in brain electrical activity.

#### 3.3.2 Spectral domain features

Frequency domain features characterize the oscillatory components of EEG signals, revealing spectral power distributions across clinically relevant frequency bands that reflect various cognitive states and neural processing mechanisms. These features are extracted through spectral analysis techniques that decompose the time-domain signal into its constituent frequency components.

Power spectral density estimation using Welch’s method

This study utilizes Welch’s method to compute the power spectral density (PSD) of EEG signals. This approach provides a robust spectral estimate by reducing noise through segment averaging:

(15) $$P(f)=\frac{1}{K}\sum_{i=1}^{K}P_{i}(f)$$

The power within clinically relevant frequency bands (delta, theta, alpha, beta, and gamma) is calculated by averaging spectral densities within each band’s frequency range, where $P_{i}(f)$ Represents the periodogram of the i-th segment:

(16) $$P_{i}(f)=\frac{1}{N}|\sum_{n=0}^{N-1}w(n)x_{i}(n)e^{-j2\pi fn/N}{|}^{2}$$

Here, $x_{i}$ is the i-th segment of the signal, w(n) The window function is typically the Hanning window, and N represents the segment length.

Frequency Band Analysis

The PSD is partitioned into clinically relevant frequency bands, each associated with specific neurophysiological processes:

(17) $$P_{\text{band }}=\frac{1}{\left|F_{\text{band }}\right|}\sum_{f\in F_{\text{band }}}P(f)$$

Where $F_{\text{band }}$ represents the set of frequencies within a specific band.

The spectral analysis focuses on extracting features from five clinically relevant frequency bands: delta (0.5–4 Hz), theta (4–8 Hz), alpha (8–12 Hz), beta (12–30 Hz), and gamma (30–45 Hz). We employ Welch’s method to compute the power spectral density for each frequency band, providing robust estimates of the signal’s frequency content by averaging multiple overlapping segments.

**Figure 3:**
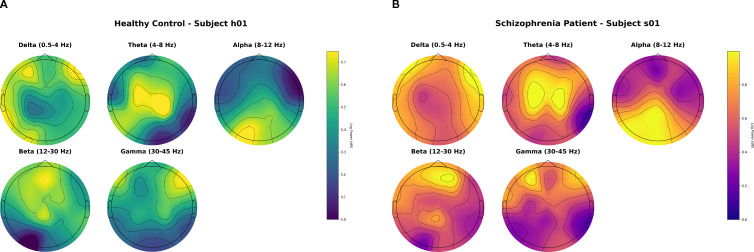
EEG power (Healthy control). **(a)** schizophrenia patient **(b)** Healthy control.

The topographic EEG maps in **Figures 3A, B** reveal significant neurophysiological differences between healthy controls and schizophrenia patients. The schizophrenia patient shows increased spectral power in the delta band (0.5–4 Hz), especially in lateral regions, indicating cortical hypoactivation commonly seen in the disorder. Their theta band (4–8 Hz) displays a distinctive bifocal frontal “butterfly” pattern, contrasting with the more central distribution seen in healthy subjects. This excess frontal slow-wave activity correlates with cognitive deficits typically observed in schizophrenia. Alpha rhythms (8–12 Hz) show perhaps the most notable difference: the healthy subject exhibits the normal posterior-dominant rhythm, while the patient demonstrates a disrupted alpha distribution with reduced posterior activity, reflecting disturbed resting-state networks. Beta oscillations (12–30 Hz) in the patient appear more focal and intense in frontal regions, suggesting altered cortical excitability and inhibitory function. The gamma band (30–45 Hz) presents asymmetrical distribution patterns in the patient, in contrast to the more balanced activity in the control subject, which is consistent with disrupted information processing and cognitive integration. The patient’s EEG shows higher absolute power values, more asymmetrical distribution patterns, and focal irregularities across multiple frequency bands. These findings align with established neurophysiological markers of schizophrenia and may reflect underlying thalamocortical connectivity disruptions and neurotransmitter imbalances central to the disorder’s pathophysiology.

### 3.4 Feature selection

We implemented a comprehensive feature selection approach to identify the most discriminative EEG characteristics for the detection of schizophrenia. Starting with 171 EEG-derived features extracted from bandpass-filtered signals (0.5–45 Hz), we employed a multi-method selection framework combining statistical significance testing, information theory metrics, and ensemble learning techniques to ensure robust feature identification.

#### 3.4.1 F-score method (ANOVA)

The F-Score method, based on Analysis of Variance (ANOVA), evaluates features by quantifying the ratio between between-class and within-class variability. For each feature, this approach calculates:

(18) $$F=\frac{\text{ Between-group variability }}{\text{ Within-group variability }}=\frac{\sum_{i=1}^{k}n_{i}{\left({\bar{x}}_{i}-\bar{x}\right)}^{2}/(k-1)}{\sum_{i=1}^{k}\sum_{j=1}^{n_{i}}{\left(x_{ij}-{\bar{x}}_{i}\right)}^{2}/(N-k)}$$

Where k is the number of classes, $n_{i}$ is the number of samples in class *i*, ${\bar{x}}_{i}$ is the mean of feature values in class *i*, $\bar{x}$ is the overall mean, and N is the total number of samples. A higher F-score indicates greater discriminative power, as it proposes the feature exhibits significant differences between classes while remaining relatively consistent within each class. The implementation selects features with p-values below 0.05, indicating a statistically significant relationship with the target classification.

#### 3.4.2 Mutual information

Mutual Information provides a more general, non-linear measure of dependence between features and target classes. This information-theoretic approach quantifies how much uncertainty about the class label is reduced by knowing a particular feature’s value:

(19) $$I(X;Y)=\sum_{y\in Y}\sum_{x\in X}p(x,y)\log(\frac{p(x,y)}{p(x)p(y)})$$

Unlike correlation-based methods that only capture linear relationships, mutual information detects any statistical dependence. The algorithm ranks features based on their mutual information scores. It selects the top performers, effectively identifying those that provide the most informative content for classification, regardless of whether the relationship is linear.

#### 3.4.3 Random forest feature importance

Random Forest feature importance provides an additional perspective by evaluating the contribution of each feature to prediction accuracy within an ensemble of decision trees. For a feature $X_{j}$ importance is computed as:

(20) $$I(X_{j})=\frac{1}{N_{\text{trees }}}\sum_{T}\sum_{t\in T:v(t)=j}p(t)\text{Δ}i(t)$$

Where p(t) is the proportion of samples reaching node t, and Δi(t)  is the impurity decrease:

(21) $$\text{Δ}i(t)=i(t)-\frac{N_{\text{left }}}{N_{t}}i(t_{\text{left }})-\frac{N_{\text{right }}}{N_{t}}i(t_{\text{right }})$$

Typically, Gini impurity is used:

(22) $$i(t)=\sum_{k}p_{k}(1-p_{k})$$

Features that consistently create pure child nodes (where samples predominantly belong to a single class) receive higher importance scores. The implementation selects features with above-average importance, focusing on those that demonstrably improve classification performance within the forest structure.

#### 3.4.4 Ensemble feature selection approach

The combined ensemble approach to feature selection leverages the complementary strengths of these three methods. Initially, it identifies features in the intersection of all methods—those consistently selected across different criteria:

(23) $$S_{\text{initial }}=S_{1}\cap S_{2}\cap S_{3}$$

This conservative approach prioritizes robustness and reliability. If this intersection yields fewer features than a predefined minimum threshold T_{min}, the algorithm progressively incorporates additional features from the union based on selection frequency across methods:

(24) $$S_{\text{final }}=S_{\text{initial }}\cup \{f_{1},f_{2},\ldots ,f_{k}\}$$

Where, $k=T_{min}-|S_{\text{initial }}|$ is the number of additional features needed? $f_{1},f_{2},\ldots ,f_{k}$​ are the k features with the highest Count(f) values from $\left(S_{\text{union }}-S_{\text{initial }}\right)$Count(f) is defined as:

(25) $$Count(f)=\sum_{i=1}^{3}1_{f\in S_{i}}$$

This counts the number of methods (from 1 to 3) that selected feature f, where 1 is the indicator function. This adaptive strategy balances specificity with sensitivity, ensuring the selection of a sufficient number of discriminative features while minimizing the risk of overfitting or including noisy, uninformative variables.

Our selection process employed three complementary methods. The initial F-score analysis through one-way ANOVA testing identified 76 significant features (p < 0.05), capturing temporal characteristics including standard deviations, skewness, and kurtosis measurements. The mutual information assessment expanded the selection to 85 features, particularly highlighting spectral power characteristics derived through Welch’s method, while measuring statistical dependence between features and class labels without assuming linear relationships. Additionally, Random Forest analysis with 100 estimators identified 57 features based on Gini impurity reduction, effectively capturing both linear and nonlinear patterns in the EEG data.

The overlap analysis among these methods offered valuable insights into feature importance. Mutual information and random forest methods showed the highest agreement with 45 shared features, followed by F-score and mutual information with 41 features, while F-score and random forest shared 31 features. Most notably, 27 features were consistently identified across all three approaches, forming our final feature set. These consensus features include both temporal and spectral characteristics, such as standard deviations, kurtosis, and skewness from various brain regions in the temporal domain, along with power distributions across alpha (8–12 Hz), beta (12–30 Hz), gamma (30–45 Hz), delta (0.5–4 Hz), and theta (4–8 Hz) frequency bands from central, parietal, occipital, and temporal regions.

This comprehensive selection process ensures our classification models utilize the most informative EEG characteristics while minimizing computational complexity and reducing the impact of noise and redundant information. The selected features effectively capture both time-domain signal variations and frequency-specific power distributions, providing a robust foundation for schizophrenia detection.

### 3.5 LSTM-based model

**Figure 4:**
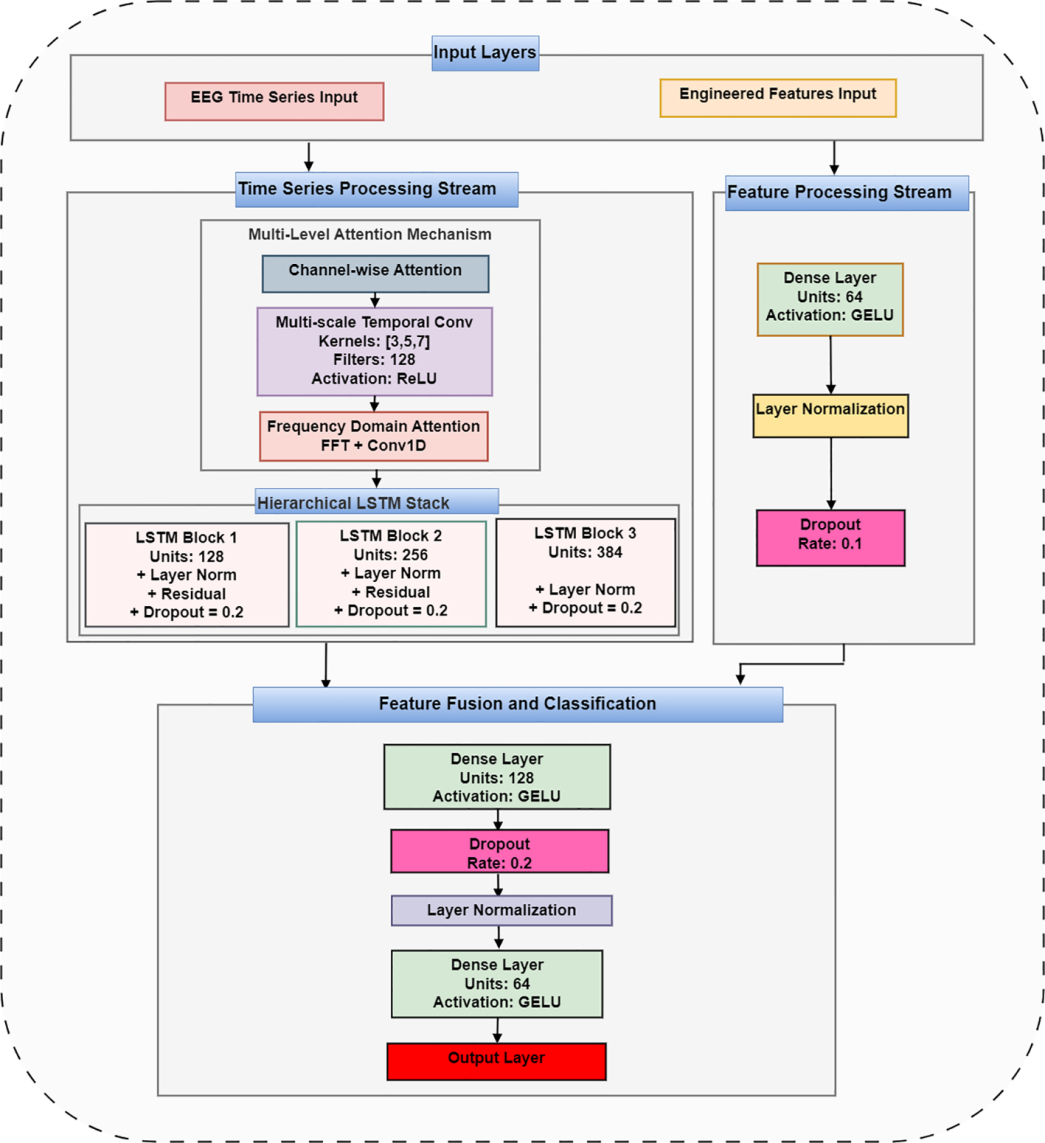
Shows the LSTM-based Model for schizophrenia detection.

The LSTM-based model integrates Long Short-Term Memory (LSTM) networks, attention mechanisms, and multi-scale temporal convolutional blocks. This design recognizes that schizophrenia-related EEG biomarkers are encoded within both the temporal evolution of neural oscillations and frequency-specific power distributions. The architecture employs a dual-pathway system, comprising a time-series processing branch for analyzing raw EEG epochs and a feature processing branch for pre-computed statistical descriptors. **Figure 4** shows the LSTM-based Model for schizophrenia detection.

#### 3.5.1 Time-series feature extraction branch

This branch processes raw temporal EEG data to identify subtle electrophysiological patterns that may be indicative of schizophrenia.

*a)Input stage:*

The initial stage receives pre-segmented EEG epochs as tensors containing the temporal data points and channel information. Each input represents discrete time points sampled at 250 Hz across the 19 EEG electrodes positioned according to the standard 10–20 system.

*b)Channel-wise attention allocation module:*

A channel attention layer at the forefront implements a learnable channel-specific weighting mechanism. Using fully connected layers with ReLU and Sigmoid activations, this module adaptively emphasizes channels with higher signal-to-noise ratios and greater relevance to the classification task, mirroring neurophysiological principles of spatially varying biomarker expression.

*c)Multi-scale temporal convolutional network (MSTCN):*

Following channel attention, a Temporal Conv Block implements parallel one-dimensional convolutional filters with diverse kernel sizes)3, 5, 7(. This multi-resolution approach extracts temporal features across scales—from high-frequency transient events to broader low-frequency oscillatory trends. Outputs from these parallel pathways are concatenated, transformed via 1×1 convolution, and normalized. A residual connection mitigates vanishing gradients, facilitating deeper network training.

*d)Dropout regularization layer:*

A Dropout layer (rate=0.1) after the MSTCN mitigates overfitting by randomly deactivating neuronal activations during training, promoting robust feature learning and improved generalization.

*e)Frequency-domain attention allocation layer:*

A Frequency Attention layer selectively emphasizes discriminative frequency bands for the detection of schizophrenia. Implemented using 1D convolutions and dense layers with Sigmoid activation, this layer learns to attend to frequency components (delta: 0.5–4 Hz, theta: 4–8 Hz, alpha: 8–12 Hz, beta: 12–30 Hz, gamma: 30–45 Hz) most salient for differentiating between healthy controls and patients.

*f)Recurrent feature extraction network:*

Refined temporal-spectral features are processed by cascaded LSTM Blocks. Each block comprises a unidirectional LSTM layer, layer normalization, and a residual connection. To facilitate hierarchical feature abstraction, LSTM units progressively increase across successive blocks (embed_dim × (i+1), starting with 128 units), capturing increasingly complex temporal patterns. Intermittent Dropout layers (rate=0.3) enhance robustness against overfitting.

*g) Time-series branch output:*

The final LSTM block produces a high-level feature vector encoding learned temporal-spectral dynamics for subsequent fusion with the feature branch output.

#### 3.5.2 Statistical feature assimilation branch

This parallel branch incorporates pre-calculated statistical features from EEG epochs, providing complementary clinical information.

*a) Input stage:*

This branch receives a pre-computed statistical feature vector containing time-domain metrics (mean, standard deviation, skewness, kurtosis) and frequency-domain power within canonical EEG bands.

*b) Feature transformation network:*

The statistical feature vector passes through a multi-layer perceptron with GELU activation functions, layer normalization, and Dropout (rate=0.1). This network transforms the input to a dimension of embed_dim/2 (64 units), learning a refined, lower-dimensional embedding of the statistical feature space and capturing potential synergistic relationships between descriptors.

#### 3.5.3 Hybrid feature fusion and classification layer

In the final stage, the Feature Concatenation Classification Network (FCCN) combines and processes the extracted features from both branches to produce the classification output.

*a)Feature concatenation:*

Output vectors from both branches are concatenated to create a unified hybrid representation, leveraging both learned deep representations and interpretable statistical summaries.

*b)Classification network and output stage:*

The concatenated vector passes through dense layers (embed_dim=128 units followed by embed_dim/2 = 64 units) with GELU activation, Dropout (rate=0.2), and layer normalization (epsilon=1e-6). A final dense layer with sigmoid activation probability representing the likelihood of schizophrenia. Binary classification is achieved by thresholding at 0.5.

The LSTM-based model synergistically combines recurrent networks, attention mechanisms, and multi-scale convolutions to capture both temporal evolution and frequency-specific characteristics. Channel and frequency attention modules focus computational resources on salient signal aspects, enhancing interpretability and efficiency. The MSTCN and LSTM layers capture short-term and long-term dependencies crucial for discerning subtle biomarkers. Pre-computed statistical features provide a clinically relevant complementary information stream.

### 3.6 Transformer-based model

**Figure 5:**
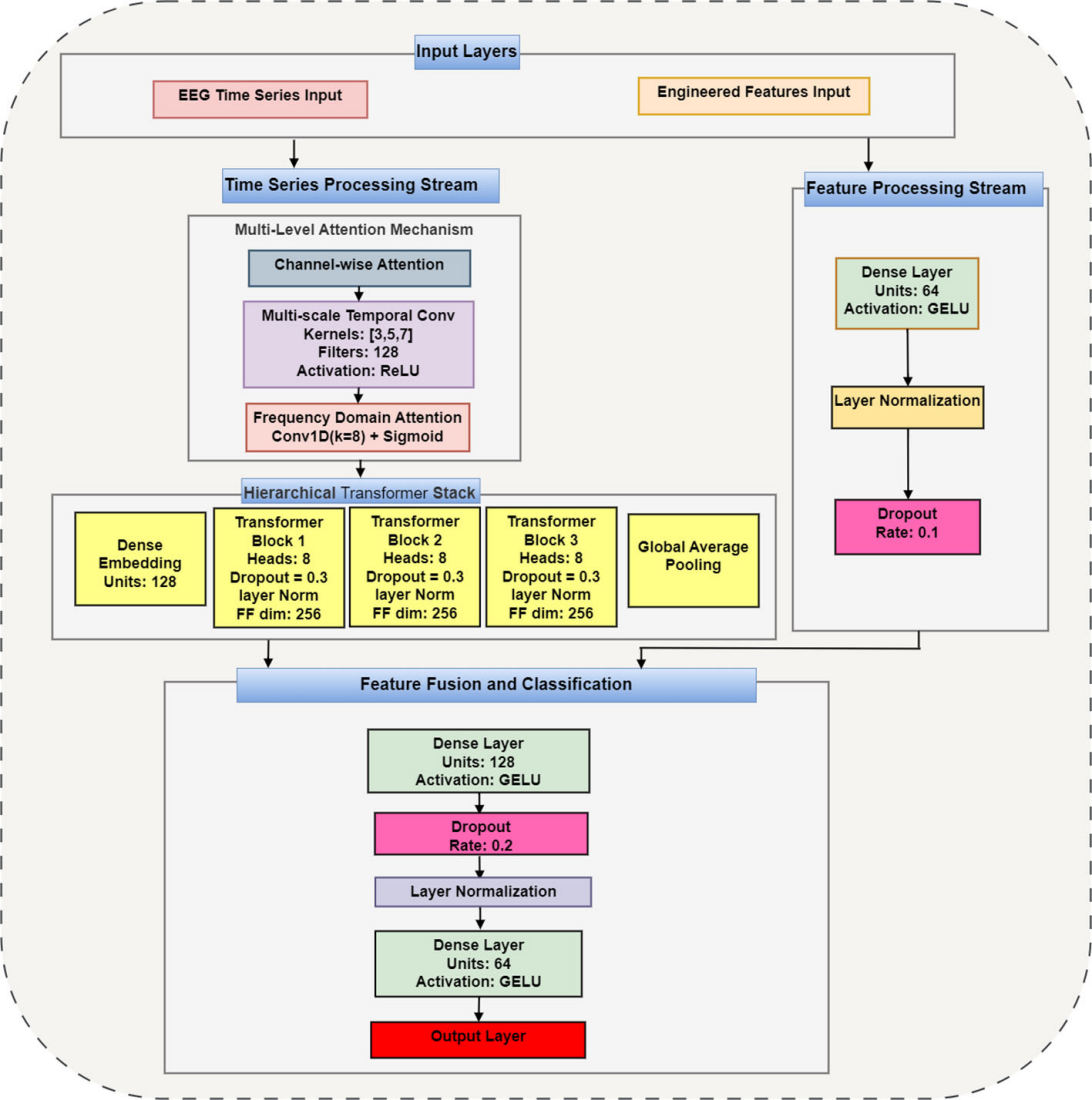
Shows the transformer-based model for schizophrenia detection.

The Transformer-based model utilizes Transformer networks to capture long-range dependencies and global contextual information. This architecture maintains the dual-branch structure but replaces recurrent LSTM layers with Transformer encoder blocks. **Figure 5** shows the Transformer-based Model for schizophrenia detection.

#### 3.6.1 Time-series feature extraction branch

The Time-Series Feature Extraction Branch processes raw EEG signals to identify patterns indicative of schizophrenia.

*a)Initial processing layers:*

Input, Channel-Wise Attention, MSTCN, Dropout, and Frequency-Domain Attention layers are identical to those in the LSTM-based model, ensuring comparability in early feature extraction.

*b)Transformer encoder blocks*

Following frequency attention, features are stacked in a series of Transformer Blocks. Each block incorporates multi-head self-attention mechanisms (num_heads=8, key_dim=embed_dim), feed-forward networks (Dense layers with GELU activation, feed-forward neural network (ff_dim=256), and layer normalization (epsilon=1e-6). This architecture captures non-local dependencies and long-range interactions within the EEG time-series, potentially uncovering subtle, distributed patterns indicative of schizophrenia. Strategic Dropout layers (rate=0.3) enhance robustness, as shown in **Figure 5**.

*c)Global feature aggregation:*

Unlike the LSTM-based model, the final Transformer Block output passes through a GlobalAveragePooling1D layer, reducing the temporal dimension to a fixed-length vector that summarizes the learned temporal context.

*d)Time-series branch output:*

The pooling layer output serves as the feature vector encapsulating globally contextualized EEG representations.

#### 3.6.2 Statistical feature assimilation branch

This branch remains identical to its counterpart in the LSTM-based model, ensuring consistency in statistical feature processing across architectures.

#### 3.6.3 Hybrid feature fusion and classification layer

Feature concatenation and classification stages mirror those in the LSTM-based model, maintaining comparability in final classification while focusing evaluation on the underlying time-series modeling approach.

The Transformer-based model addresses the hypothesis that schizophrenia-related EEG abnormalities manifest as subtle, distributed patterns requiring global context-aware modeling. Transformer blocks explicitly capture long-range dependencies and global contextual information, reflecting evidence of altered global brain network dynamics in schizophrenia. Retaining attention mechanisms, MSTCN, and statistical feature integration ensures benefits from feature selection, multi-scale temporal extraction, and clinically relevant data while exploiting the powerful global context modeling capabilities of Transformers.

## 4 Experimental results

In this section, we present a comprehensive evaluation of our proposed deep learning approaches for EEG-based schizophrenia detection. Through extensive testing and validation, we demonstrate the effectiveness of both Transformer and LSTM architectures in distinguishing between schizophrenia patients and healthy controls.

### 4.1 Experimental environment setup

The implementation and evaluation of our proposed deep learning methods were carried out in a controlled experimental setting to ensure reproducibility and accurate performance assessment. We used high-performance computing resources with Google Colab GPUs, specifically optimized for the efficient training of complex neural networks. The computing setup was configured to manage both the extensive preprocessing needs and the computational demands of our dual model comparison.

### 4.2 Evaluation metrics

We developed a thorough evaluation framework to assess our models’ performance in EEG-based schizophrenia detection. Our assessment uses multiple complementary metrics, including accuracy, sensitivity, specificity, F1-score, and Area Under the ROC Curve (AUC). These metrics are selected to give detailed insights into different aspects of clinical usefulness, from overall prediction accuracy to specific discriminative abilities. Both models were tested using 5-fold cross-validation to ensure reliable performance evaluation and validation of their clinical potential. Our models were trained with the hyperparameters listed in **Table 3**.

**Table 3:** Training hyperparameters.

Hyperparameter	Value	Description
Learning Rate	1e-4	Initial learning rate for Adam optimizer
Batch Size	64	Number of samples per training batch
Max Epochs	100	Maximum number of training epochs
Early Stopping Patience	5	Epochs to wait before early stopping
Learning Rate Reduction Factor	0.5	Factor to reduce LR on plateau
Learning Rate Patience	3	Epochs to wait before LR reduction

(26) $$\text{Accuracy }=\frac{\text{True Positives (TP)}+\text{True Negatives }(\text{TN})}{\text{True Positives (TP)}+\text{True Negatives }(\text{TN})+\text{False Positives }(\text{FP})+\text{False Negatives }}$$

(27) $$Specificity=\frac{\;True\;Negative\;}{\;True\;Negative+False\;Negative\;}$$

(28) $$Sensitivity=\frac{\;True\;Positive\;}{\;True\;Positive+False\;Positive\;}$$

(29) $$\text{F}1-\text{Score }=2\times \frac{\text{ Precision}\times \text{Recall }}{\text{ Precision}+\text{Recall }}$$

Area under the curve (AUC)

(30) $$\text{TPR}=\frac{\text{TP}}{\text{TP}+\text{FN}}$$

(31) $$\text{FPR}=\frac{\text{FP}}{\text{FP}+\text{TN}}$$

### 4.3 Model performance analysis using the cross-validation method

This section assesses the performance of two advanced deep learning models—LSTM-based and Transformer-based—that are designed to classify EEG data to differentiate schizophrenia patients from healthy controls. Using 5-fold cross-validation, we evaluate each model’s performance using key metrics: accuracy, sensitivity, specificity, F1 score, and AUC.

#### 4.3.1 LSTM-based model

The LSTM model performs well in classifying EEG data to differentiate schizophrenia patients from healthy controls, as shown in **Table 4**, with a mean accuracy of 95.77% during testing. The mean accuracy loss across five folds is 0.16. In the training phase, the LSTM model achieved 97.30% accuracy, with a loss of 0.07.

**Table 4:** Summary result for the LSTM-based model.

	Train_accuracy	Train_loss	Test_accuracy	Test_loss	Accuracy	Sensitivity	Specificity	F1
Fold_1	98.26	0.05	97.09	0.13	97.07	96.11	98.01	97.01
Fold_2	98.17	0.05	96.63	0.13	96.77	96.02	97.49	96.71
Fold_3	93.36	0.17	90.46	0.28	91.82	95.82	87.90	92.07
Fold_4	98.88	0.03	97.47	0.11	96.99	96.02	97.93	96.93
Fold_5	97.85	0.06	96.16	0.14	96.18	97.44	94.95	96.20
Mean	97.30	0.07	95.56	0.16	95.77	96.28	95.26	95.78
Std	2.00	0.05	2.59	0.06	1.99	0.59	3.84	1.88

**Figure 6:**
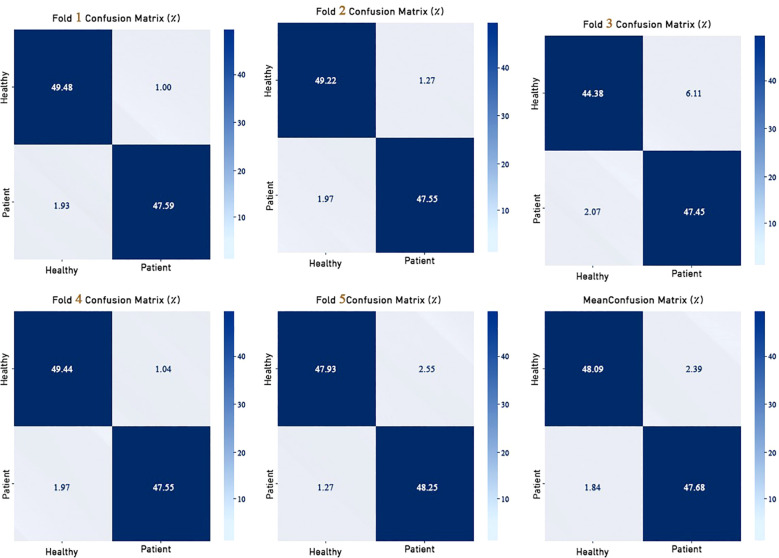
Confusion matrix for the LSTM-based model.

Used to assess a binary classification model separating between “Healthy” and “Patient” people, **Figure 6** presents mean confusion matrixes along with five cross-validation folds’ confusion matrices. Presented as percentages, each matrix shows classification performance; high diagonal values indicate great classification accuracy. Off-diagonal cells indicate misclassifications; diagonal cells denote genuine positives—Healthy projected as Healthy—and true negatives—Patient predicted as Patient. With average accurate classification rates of 48.09% for Healthy and 47.68% for Patients, and low misclassification rates (1.84% and 2.39%), the model maintains steady performance throughout all folds. This shows the model’s strength and balanced performance across both categories.

**Figure 7:**
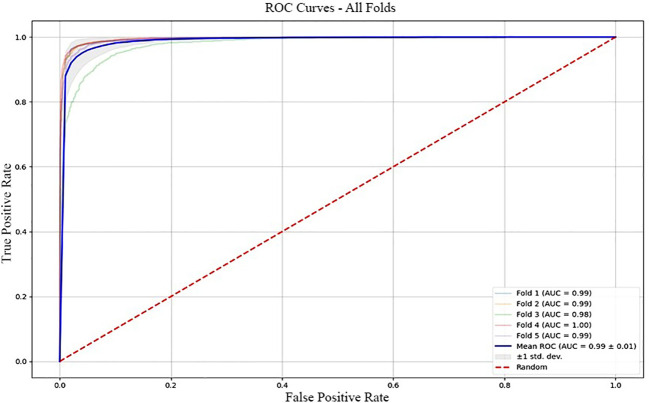
ROC curve for the LSTM-based model.

**Figure 7** displays ROC curves for five cross-validation folds and an average curve. The curve shows the trade-off between the True Positive Rate and the False Positive Rate at different threshold levels. With high sensitivity and few false positives, all folding curves are tightly clustered near the top-left corner. The AUC values for the folds range from 0.98 to 1.00, indicating excellent and consistent model performance, with an average AUC of 0.99 ± 0.01. Highlighting the model’s superiority, the red dashed line represents a random classifier (AUC = 0.5). The thin shaded area around the mean ROC curve indicates slight variation, further confirming the model’s reliability and robustness.

**Figure 8:**
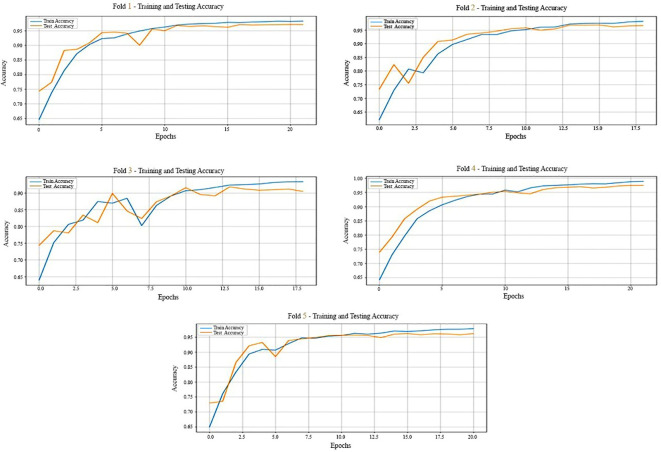
Accuracy performance of LSTM model.

**Figure 8** shows the model’s learning behavior over epochs, displaying training and testing accuracy curves for five folds in a cross-validation experiment. With final accuracies usually above 96%, the training and testing accuracies in each fold consistently increase and converge, indicating strong learning and generalization. Most folds show a close match between the training and testing curves, indicating minimal overfitting; only small differences are seen in Fold 3.

**Figure 9:**
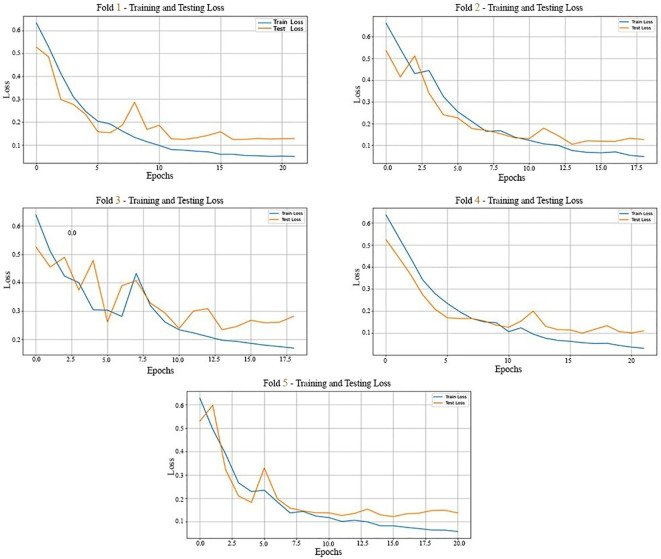
Loss performance of LSTM model.

**Figure 9** shows five-line graphs, each showing the training and testing loss of a LSTM model over a range of epochs from Fold 1 through Fold 5. Each graph’s x-axis represents the number of epochs, ranging from 0 to 20, while the y-axis displays the loss, ranging from 0.0 to 0.6. Each fold has two lines drawn: an orange line for the testing loss and a blue line for the training loss. Though there are significant variations, especially in Folds 3 and 5, the training loss usually drops with time across all folds, suggesting the model is learning from the training data. The test loss usually decreases as well.

#### 4.3.2 Transformer-based model

The transformer model performs exceptionally well in distinguishing schizophrenia patients from healthy controls using EEG data, with an average accuracy of 99.56% (± 0.15%), sensitivity of 98.12% (± 0.40%), specificity of 98.11% (± 0.54%), F1 score of 98.09% (± 0.24%), and AUC of 99.84% (± 0.03%) across five-fold cross-validation. Its high accuracy and consistency make it a leading tool for EEG-based diagnostic support. **Table 5** summarizes the performance metrics of the transformer model for classifying EEG data to differentiate schizophrenia patients from healthy controls, based on five-fold cross-validation. **Figure 8** displays the confusion matrix, while **Figure 9** presents the ROC curve for the LSTM-based model.

**Table 5:** Summary result for the TF model.

Fold	Train_accuracy	Train_loss	Test_accuracy	Test_loss	Accuracy	Sensitivity	Specificity	F1
Fold_1	99.58	0.01	98.19	0.06	98.05	97.53	98.57	98.02
Fold_2	99.47	0.02	98.21	0.06	98.13	97.89	98.37	98.11
Fold_3	99.44	0.02	97.85	0.07	97.81	98.09	97.53	97.80
Fold_4	99.84	0.01	98.49	0.06	98.55	98.42	98.69	98.54
Fold_5	99.45	0.02	98.23	0.06	98.01	98.66	97.37	98.01
Mean	99.56	0.01	98.20	0.06	98.11	98.12	98.11	98.09
Std	0.15	0.00	0.20	0.00	0.25	0.40	0.54	0.24

**Figure 10:**
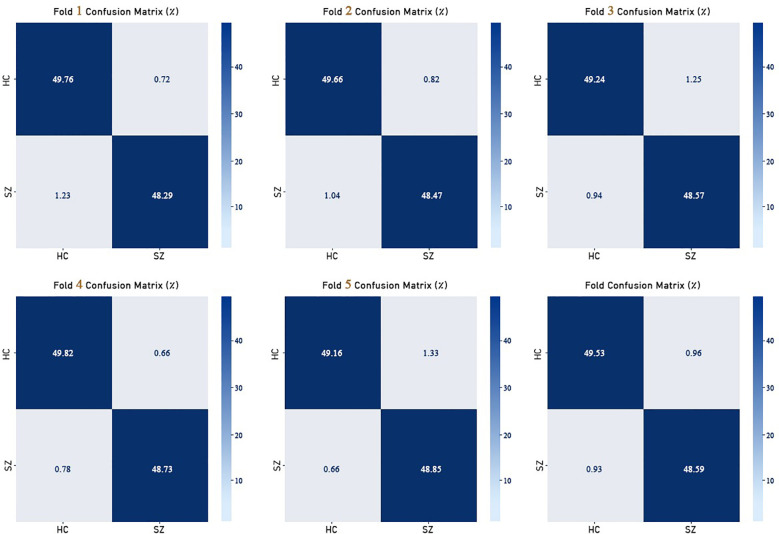
Confusion matrix for LSTM based model.

**Figure 10** shows the confusion matrix of the TF model. Where HC probably stands for “Healthy Control” and SZ for “Schizophrenia” or a related condition, each matrix is a 2x2 grid with the true labels (HC and SZ) on both axes. While the off-diagonal cells show misclassifications, the diagonal cells (top-left and bottom-right) show accurate predictions. With misclassification rates of 0.72% and 1.22%, the model for Fold 1 accurately detects 48.79% of HC and 48.29% of SZ. With 0.82% and 1.04% errors, Fold 2 reveals 48.66% for HC and 48.47% for SZ. With 24.14% for HC and 48.57% for SZ, Fold 3 is less accurate and has more mistakes of 1.25% and 0.94%. With 48.82% for HC and 48.73% for SZ, Fold 4 shows some improvement, having 0.66% and 0.78% errors, respectively. With 1.33% and 0.66% errors, Fold 5 reports 48.16% for HC and 48.45% for SZ. Averaging these findings, the Mean Confusion Matrix reveals 48.51% for HC and 48.50% for SZ, with misclassification rates of 0.96% and 0.93%. Reflecting the percentage values, the color intensity, ranging from light to dark blue, indicates deeper hues, suggesting larger percentages. A color bar on the right provides a scale from 0 to 50%.

**Figure 11:**
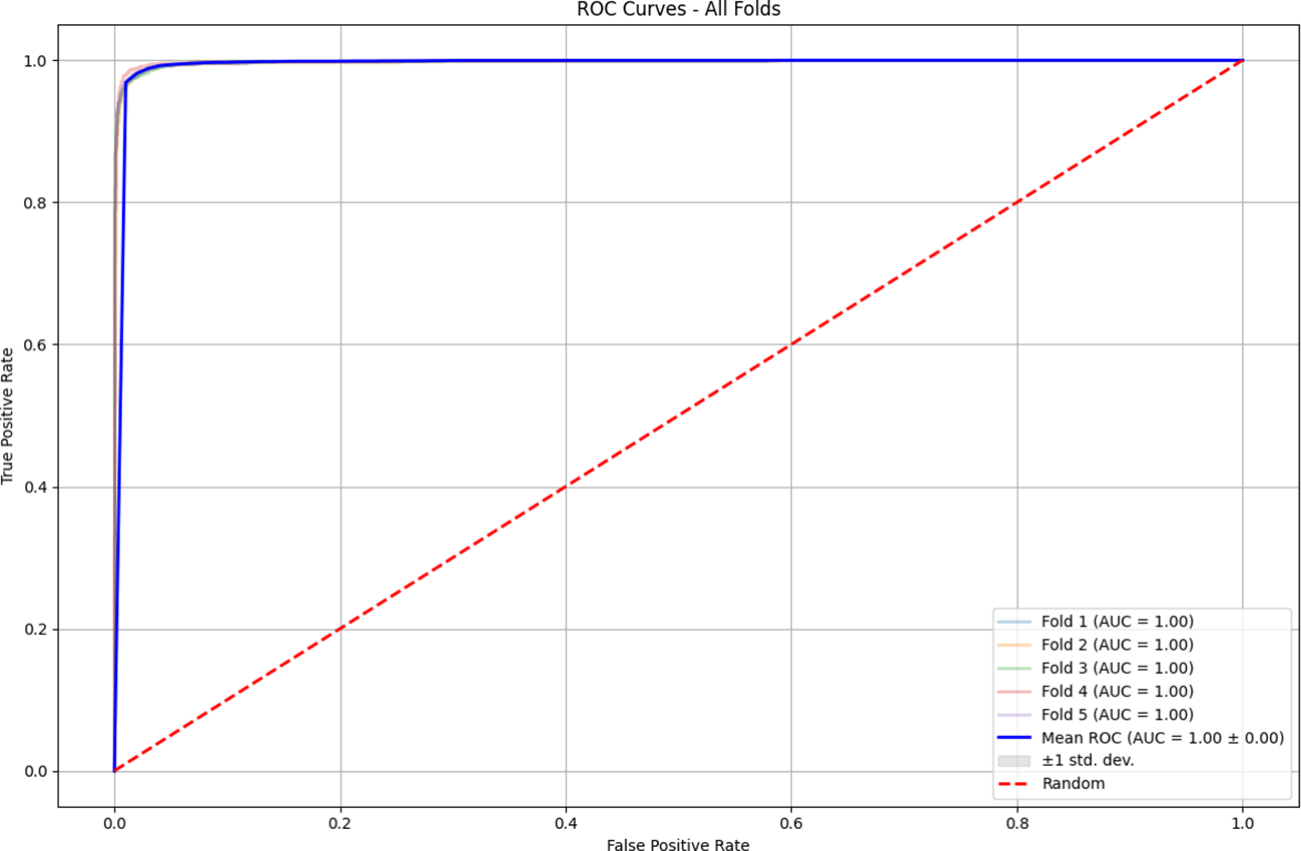
ROC curve for LSTM based model.

**Figure 11** shows an ROC curve assessing the performance of a binary classification model throughout five folds. The x-axis shows the FPR from 0.0 to 1.0, and the y-axis shows the TPR from 0.0 to 1.0 as well. Each of the five folds (Folds 1–5) demonstrates perfect classification performance, with no false positives or negatives, achieving an Area Under the Curve (AUC) of 1.00, as represented by a distinct colored line. Begin with the first fold in blue, then go on to tangerine, emerald, rose, and finally violet. Dark blue depicts the mean ROC curve, which confirms consistent performance across all folds with an AUC of 1.00 and a standard deviation of ±0.00. Running diagonally from (0,0) to (1,1), a red-dashed line labeled “Random” shows the baseline performance of a random classifier with an AUC of 0.5. Hugging the top-left corner, the ROC curves for all folds and the mean curve show that the model has a TPR of 1.0 and an FPR of 0.0, hence indicating outstanding discriminative capacity across all folds between the two classes.

**Figure 12:**
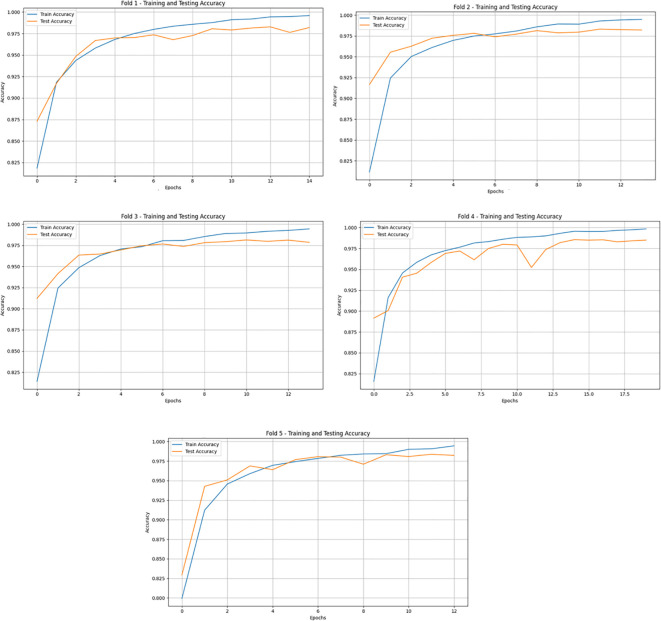
Accuracy performance of TF model.

**Figure 12** displays the training and testing accuracy across five cross-validation folds. Each subplot shows a specific fold, illustrating how accuracy progresses over training epochs. The TF model demonstrates a steady increase in both training and testing accuracy, indicating effective learning and good generalization across all folds. Starting at around 82–87%, the accuracy gradually improves as training continues; most folds reach a testing accuracy of 97%. The close alignment of the training and testing accuracy curves also suggests minimal overfitting. This consistent performance across all folds emphasizes the model’s reliability and robustness in classification tasks.

**Figure 13:**
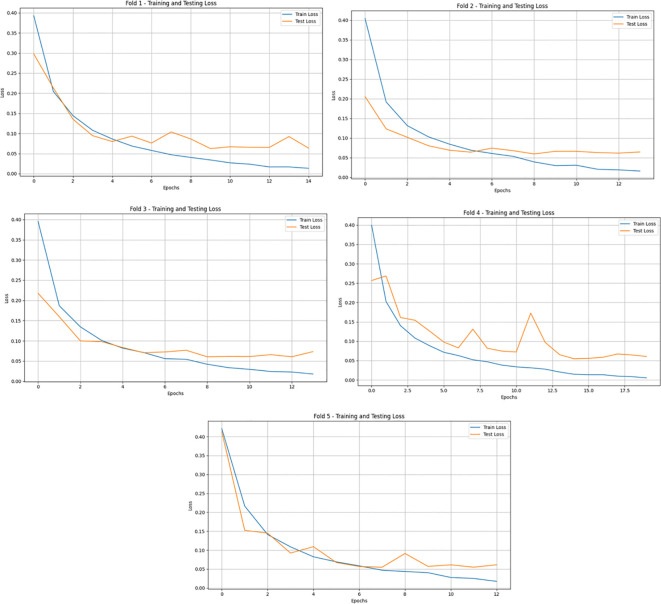
Loss of performance of TF model.

**Figure 13** shows the training and testing loss curves for each of the five cross-validation folds. The model demonstrates efficient learning and decreasing error across epochs, with a steady and rapid decline in both training and testing loss in all folds. Starting high—around 0.35 to 0.40—the initial loss drops significantly toward levels of 0.02 or lower by the later epochs. Most folds exhibit little difference between training and testing loss, indicating the model generalizes well without significant overfitting. These results confirm that the TensorFlow model converges strongly, reducing loss and maintaining consistent performance across all validation splits.

### 4.4 Performance analysis of the proposed model using the LOSO method

In this experiment, we used a features-baseline method to extract features from the EEG dataset. For each one-second epoch, 171 features were computed from the EEG signals, including time-domain features (mean, SD, skewness, and kurtosis) and frequency-band powers across five bands for each of the 19 channels. A hybrid feature selection approach combining mutual information, F-score, and random forest importance was used to shortlist the 27 most distinguishing features for each fold. These features were normalized for classification using a deep learning model with three layers, including layer normalization and dropout.

**Table 6** shows that the average test accuracy across all subjects using LOSO validation was 66.97% ± 22.74%. This large standard deviation indicates substantial variability between subjects, with accuracy ranging from 10.94% to 97.64% per subject. The noticeable variability suggests that while some discriminative information is captured in the statistical features, these features may not fully capture the complex temporal dynamics needed to effectively differentiate between schizophrenic patients and healthy subjects.

**Table 6:** Results of the feature-based baseline.

Participant	Group	Test accuracy (%)
h01	HC	42.05
h02	HC	48.57
h03	HC	89.67
h04	HC	51.24
h05	HC	96.83
h06	HC	56.56
h07	HC	40.44
h08	HC	89.01
h09	HC	10.94
h10	HC	54.89
h11	HC	90.71
h12	HC	63.44
h13	HC	96.99
h14	HC	52.72
s01	SZ	88.40
s02	SZ	70.92
s03	SZ	75.83
s04	SZ	32.03
s05	SZ	85.51
s06	SZ	28.11
s07	SZ	76.52
s08	SZ	62.24
s09	SZ	97.64
s10	SZ	80.24
s11	SZ	53.24
s12	SZ	81.60
s13	SZ	75.42
s14	SZ	83.36

**Figure 14:**
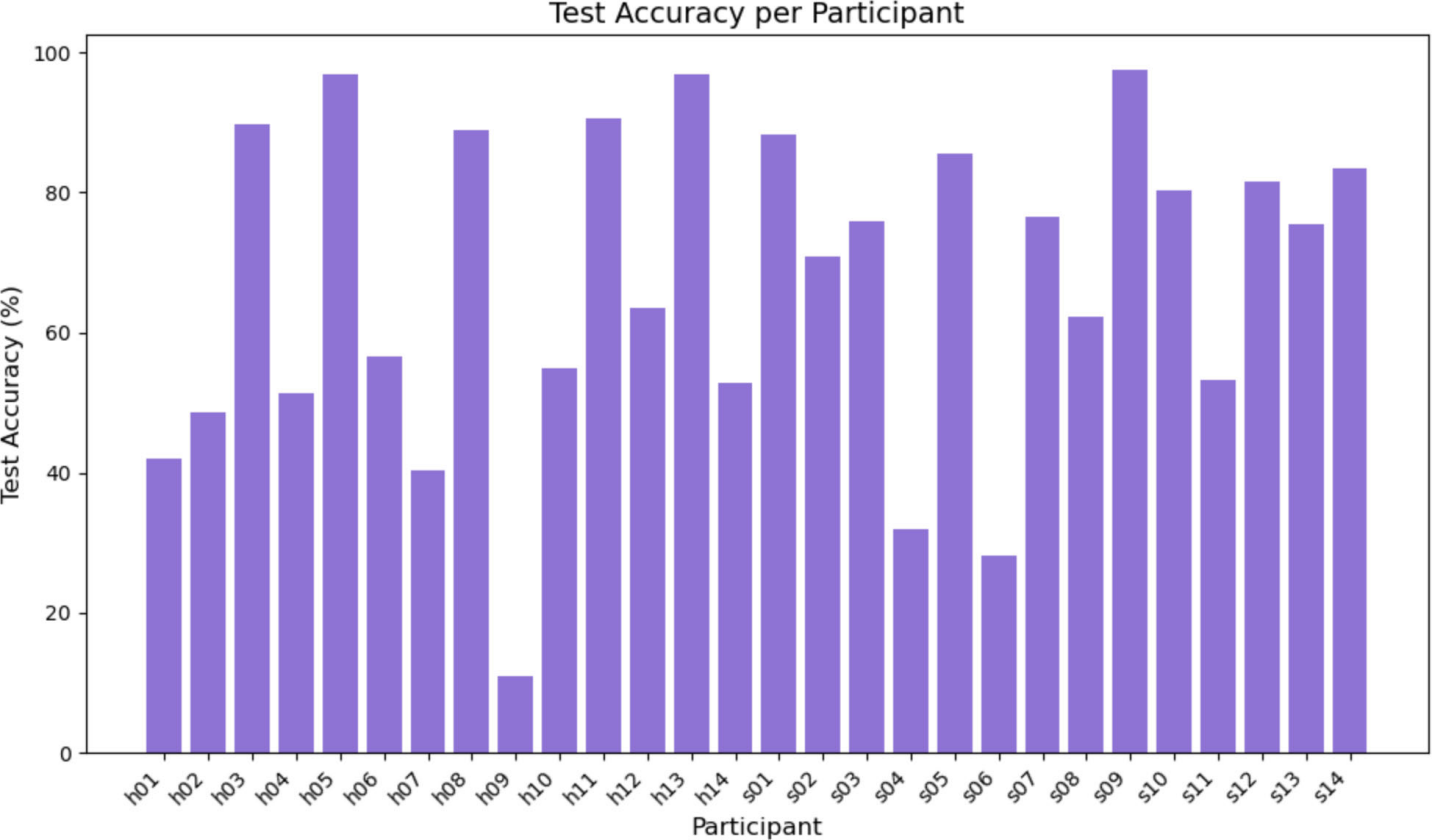
Performance based on feature-based baseline.

**Figure 14** shows the accuracy of the proposed system relative to the feature-based baseline for 28 participants, using the LOSO-CV method. Several participants achieve high accuracy (80–97%), indicating that the model effectively represents their EEG patterns. However, some participants have much lower accuracy. Specifically, participants h01, h02, h05, h07, h09, h12, h14, s02, s05, s06, s08, s11, and others show significantly lower accuracy than the rest of the cohort.

In this experiment, we used a time series model to extract features from the Sz EEG dataset. The time series transformer model was developed to test whether raw EEG features could be used to classify schizophrenia. Each one-second epoch (250 samples x 19 channels) was filtered with a band-pass filter (0.5–45 Hz), followed by a transformer model with three self-attention blocks, eight self-attention heads, and embedding dimensions set at 128.

The average accuracy of the time-series-based transformer is shown in **Table 7**. The model achieved an accuracy of 71.48% across all subjects using LOSO-CV. Classification performance varies significantly across subjects, ranging from 2.76% to 99.58%, suggesting that EEG patterns in some subjects’ data are more distinguishable than in others. Compared to the features-only model (66.97% ± 22.74%), the transformer generalizes better as a Time-series model to extract features

**Table 7:** Results based on the time series features.

Participant	Group	Test accuracy (%)
h01	HC	95.03
h02	HC	48.24
h03	HC	91.76
h04	HC	96.43
h05	HC	94.60
h06	HC	84.19
h07	HC	64.95
h08	HC	62.42
h09	HC	2.76
h10	HC	96.14
h11	HC	69.40
h12	HC	10.11
h13	HC	92.54
h14	HC	29.83
s01	SZ	91.48
s02	SZ	89.00
s03	SZ	94.19
s04	SZ	95.44
s05	SZ	74.61
s06	SZ	5.54
s07	SZ	69.61
s08	SZ	52.69
s09	SZ	99.58
s10	SZ	41.88
s11	SZ	85.15
s12	SZ	68.17
s13	SZ	97.00
s14	SZ	98.71

**Figure 15:**
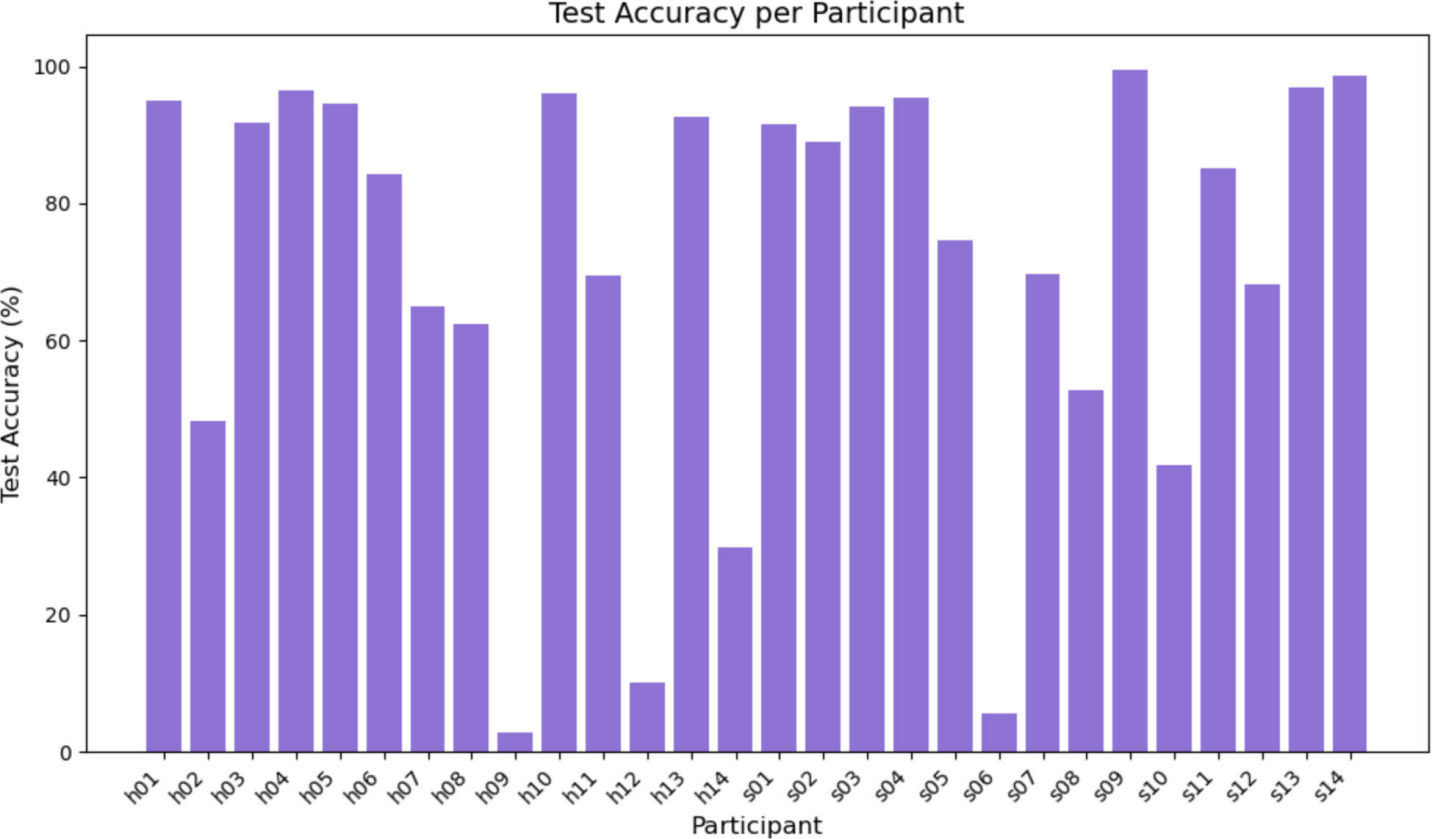
Performance based on the time series features.

**Figure 15** shows the accuracy of the proposed system based on time series features using the LOSO-CV method for diagnosing Sz. The range of accuracy indicates that EEG patterns differ significantly across subjects. Fewer participants perform very well (about 90–97%), which shows that the model works well for them, while others perform much worse, suggesting their EEG patterns are harder to interpret or vary more from one person to another. The plot demonstrates that LOSO provides a realistic, patient-independent evaluation, but it also highlights how challenging it is to develop a seizure-detection model that works for everyone.

### 4.5 Performance analysis with feature fusion in the leave-one-subject-out case

Analysis of the proposed model’s performance at the subject level revealed significant variability among individuals, reflecting natural differences in electrophysiological activity in both SZ and control groups. **Table 8** displays the classification accuracy for each subject when that individual is used as the test case. Accuracy for HC ranged from 7.40% (h09) to 97.20% (h06), while in SZ patients, it varied from 37.43% (s06) to 98.52% (s02). Notably, most individuals (75%) achieved classification accuracy above 65%, with a quarter (25%) surpassing 95%. This demonstrates considerable variation in classification results, indicating that individual neurophysiological parameters significantly impact the monitoring system’s effectiveness. While most cases offer valuable insights, some individuals still pose greater challenges for accurate classification.

**Table 8:** Performance based on fusion features.

Participant	Group	Test accuracy (%)
h01	HC	91.46
h02	HC	44.95
h03	HC	89.23
h04	HC	96.11
h05	HC	91.64
h06	HC	97.20
h07	HC	66.48
h08	HC	94.62
h09	HC	7.40
h10	HC	69.24
h11	HC	97.05
h12	HC	66.33
h13	HC	91.50
h14	HC	25.78
s01	SZ	74.56
s02	SZ	98.52
s03	SZ	95.64
s04	SZ	85.48
s05	SZ	80.56
s06	SZ	37.43
s07	SZ	97.47
s08	SZ	89.68
s09	SZ	81.77
s10	SZ	80.94
s11	SZ	83.16
s12	SZ	89.97
s13	SZ	65.81
s14	SZ	64.70

**Figure 16:**
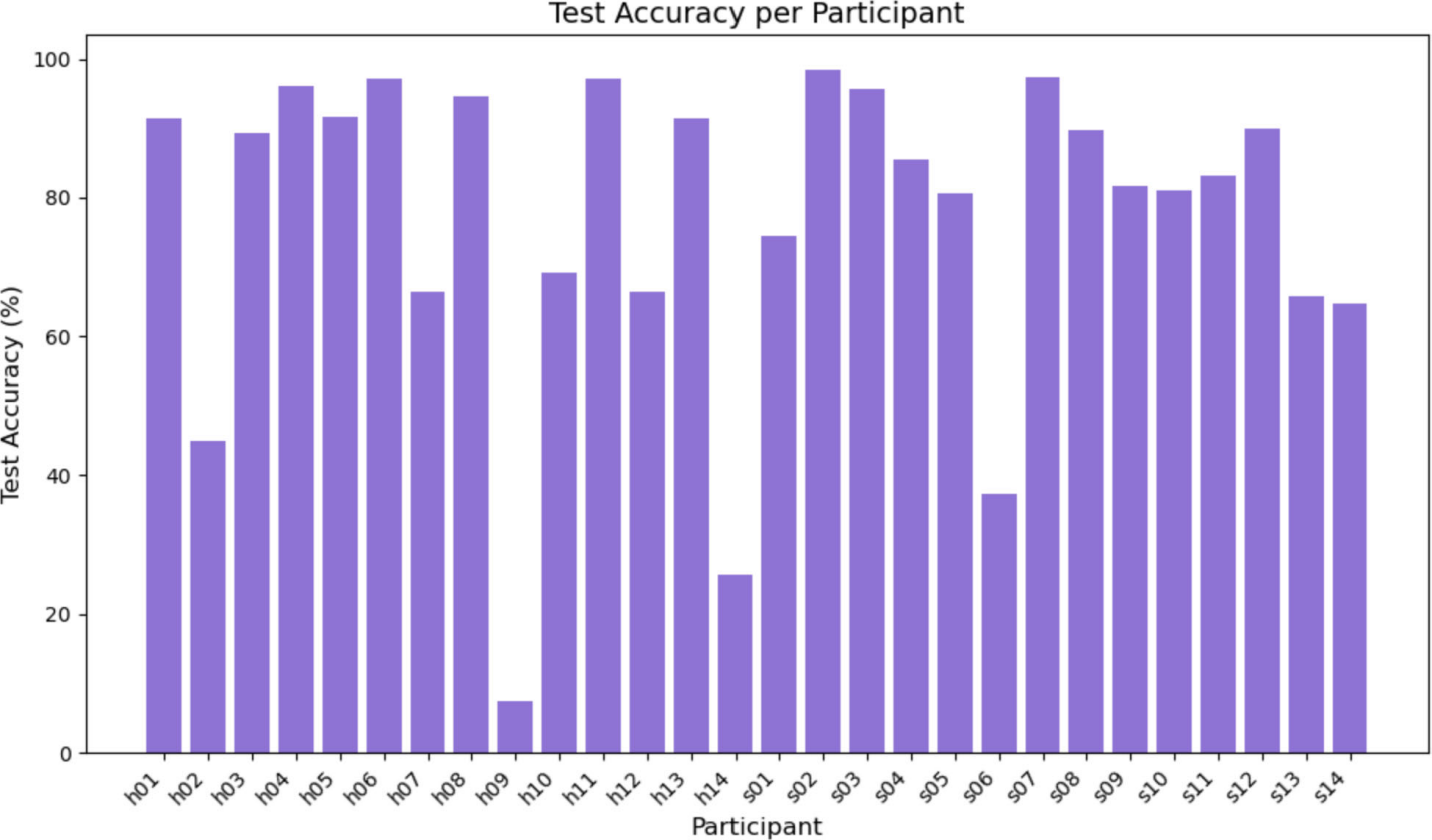
Performance on fusion features.

**Figure 16** shows a bar chart of test accuracy for each individual in the dataset. Each bar indicates how well the proposed model performed in classifying data from a single subject. The plot shows that participants had varying levels of accuracy, demonstrating that the model is consistent and can generalize when evaluated subject-by-subject. The figure also displays a DL model with fused features. Overall, the proposed model shows good generalization, with most participants achieving high scores in the 80–98% range. This indicates that everyone learned in a stable manner. However, some subjects (h02, h09, h14, s07) show significantly lower accuracies, highlighting the inherent inter-subject variability in EEG-based SZ patterns and emphasizing the challenge of subject-independent classification. This suggests that the model effectively identifies discriminative EEG features for SZ detection using LOSO validation.

## 5 Discussion

Schizophrenia is a persistent, harmful condition marked by extreme disturbances in cognition, perception, emotion, and behavior. Improving long-term clinical outcomes depends on early detection of schizophrenia as protracted untreated psychosis is linked with adverse reactions to antipsychotic drugs and progressive functional deficits. The absence of proven biomarkers depending on underlying neurobiological processes makes it still difficult, nonetheless, to identify schizophrenia correctly. The number of people suffering from mental problems has risen quickly. Therefore, more proactive studies for mental disorder diagnosis are being done. Mental disease diagnosis relies only on a psychiatrist’s diagnosis. Particularly, societies with limited access to healthcare struggle not only with early diagnosis but also with erroneous diagnosis. Improving accuracy requires broadening the scope of current studies. The proposed system enhances the accuracy and efficiency of SZ mental condition diagnosis.

Examine the proposal system based on LSTM and TF using EEG data from 28 participants. Results from this experiment were analyzed and compared with earlier studies employing various methods, including learning EEG. During LSTM learning, the observed classification accuracy was 95.56% in the TF graphs for the 28 participants. Meanwhile, the TF model achieved an average accuracy of 98.20% during testing and 99.56% during training. The Transformer’s superior performance, driven by its self-attention mechanism, highlights its ability to better capture complex EEG patterns, though this comes at a higher computational cost. While the LSTM model remains a viable option for resource-limited settings, the Transformer’s increased accuracy and consistency make it the preferred choice for maximizing diagnostic precision.

The analysis error of the LSTM model for predicting SZ is shown in **Table 9**. The LSTM model for SZ diagnosis generally performs well across multiple metrics—Area Under Curve (AUC), Matthews Correlation Coefficient (MCC), Mean Squared Error (MSE), and R² (coefficient of determination)—with most runs showing high AUC values (≈99.44%), MCC values between 0.84 and 0.94, low MSE (0.02), and high R² scores (0.74–0.90). These results indicate strong classification performance, with high true-positive and true-negative rates, minimal prediction error, and a good model fit.

**Table 9:** Analysis error of LSTM model.

AUC	MCC	MSE	R^2^
99.44	0.94	0.03	0.90
99.48	0.94	0.03	0.90
97.94	0.84	0.06	0.74
99.55	0.94	0.02	0.90
99.34	0.92	0.03	0.87
99.15	0.92	0.03	0.86
0.61	0.04	0.02	0.06

**Table 10** summarizes the analysis errors of a TF model for diagnosing SZ across multiple folds, including AUC, MCC, MSE, and R², along with their standard deviations. The AUC values are exceptionally high, ranging from 99.77% to 99.85%, with a mean of 99.82% and a standard deviation of 0.03%, indicating near-perfect ability to distinguish between SZ and non-SZ cases, since an AUC of 100% represents a perfect classifier. The MCC, a balanced measure of classification quality, ranges from 0.99.77 to 0.99.85, with a mean of 0.99.70. The MSE, which measures the average squared difference between predicted and actual values, remains consistently low, from 0.01 to 0.02, with a mean of 0.02 and a standard deviation of 0.00, indicating minimal prediction errors. Finally, the R² values, which evaluate the proportion of variance explained by the model, range from 0.93 to 0.95, with a mean of 0.94 and a standard deviation of 0.01, demonstrating that the model accounts for 94% of the data’s variability on average, thus representing a strong fit for diagnostic purposes. Overall, the TF model shows robust and consistent performance across all folds, with high accuracy, low error, and excellent explanatory power, making it highly reliable for SZ diagnosis. However, slight variations in MSE and R² suggest minor inconsistencies in predictive accuracy across folds.

**Table 10:** Analysis error of TF model.

AUC	MCC	MSE	R^2^
99.81	0.96	0.02	0.94
99.77	0.96	0.02	0.94
99.84	0.96	0.02	0.93
99.83	0.97	0.01	0.95
99.85	0.96	0.02	0.94
99.82	0.96	0.01	0.94
0.03	0.00	0.00	0.01

The results of the proposed method were compared with different existing systems, as shown in **Table 11**. It is noted that the proposed systems achieved high accuracy.

**Table 11:** Comparison with existing SZ systems.

References	Year	validation method	Models	ACC %
Ref (Hassan et al., 2023)	2023	10-fold	LR	98
Ref (Gosala et al., 2023)	2023	10-fold	DT	97
Ref (Agarwal and Singhal, 2023)	2023	Training/testing	BT	98.62
Ref (Sharma and Acharya, 2021)	2021	10-fold	KNN	97.2
Ref (Kim et al., 2021)	2020	10-fold	SVM	94.80
Ref (Das and Pachori, 2021)	2021	10-fold	SVM	98
Ref (Akbari et al., 2021)	2021	10-fold	KNN	94.80
Proposed system		K-fold and LOSO	Transformer	99.25 and 76. 95

This study employed a well-characterized dataset of 28 participants with rigorous clinical controls, including a 7-day medication washout period that ensured clean neurophysiological measurements. The controlled clinical setting provides high-quality data, though future validation in diverse clinical environments would enhance applicability. Several areas present opportunities for enhancement. Future work should apply this transformer architecture to larger, multi-site datasets to validate performance and robustness across diverse populations. Additionally, implementing individual alpha frequency detection for personalized frequency boundaries, incorporating comprehensive demographic documentation, and expanding to populations with various schizophrenia subtypes would advance the approach. Combining transformer architectures with other neuroimaging modalities could further enhance diagnostic precision. This approach establishes a foundation for next-generation EEG-based psychiatric diagnosis, with clear pathways for expanding both technical capabilities and clinical applications through larger-scale validation studies. Overall, the findings demonstrate the promise of Transformer-based deep learning approaches for EEG analysis in schizophrenia. Despite the limited dataset size, the model exhibited meaningful discriminative ability and physiological relevance. Future research involving larger, multi-center datasets and harmonized EEG acquisition protocols will be essential to confirm the clinical utility and scalability of the proposed framework.

## 6 Conclusion

This experimental analysis focused on deep learning architectures for the automatic detection of schizophrenia from EEG signals, underscoring the need for robust techniques in a clinical setting. The proposed Transformer model, applied to EEG signals collected from 28 individuals, achieved higher accuracy (98.25%) and k-fold accuracy (98.25%) than LOSO-CV (76.95%). This significant variation in results highlights the extent to which accuracy techniques influence performance interpretation. Features extracted by the proposed model revealed gamma-wave involvement in schizophrenia, as indicated by Feature selection techniques that identified a set of 27 neurophysiological features highlighting gamma waves.

The results of the LOSO analysis indicated a high degree of inter-subject variability, suggesting a high degree of realism with respect to one’s expectations in a clinical setting. Although the performance achieved at this stage can certainly classify this model as a supplementary diagnostic device rather than a standalone one, it indicates the viability of EEG-based DL solutions in the realm of psychological well-being. Some drawbacks of this model, such as a small database comprising instances from a single location and a classification task restricted to two classes, indicate a need for large-scale studies in the future.

## Data Availability

The datasets presented in this study can be found in online repositories. The names of the repository/repositories and accession number(s) can be found below: https://repod.icm.edu.pl/dataset.xhtml?persistentId=doi:10.18150/repod.0107441.
